# *Burkholderia pseudomallei* Septic Arthritis of the Knee Joint: Report of a Third Imported Case in Oman

**DOI:** 10.5339/qmj.2022.13

**Published:** 2022-03-04

**Authors:** Maya Al Salti, Mahmood Al Subhi, Amina Al-Jardani, Azza Al-Rashdi, Zayid K. Almayahi

**Affiliations:** ^1^Medical Microbiologist, Rustaq Hospital, MOH, South Batinah Governorate, Oman; ^2^Medical Microbiologist, Central Public Health Laboratories, MOH, Muscat, Oman; ^3^Medical Epidemiologist, Disease Surveillance and Control, MOH, South Batinah Governorate, Oman E-mail: almayahi96@hotmail.com

**Keywords:** Melioidosis, *Burkholderia pseudomallei*, Oman, Septic arthritis, Bacterial infections, Amoxicillin–clavulanic acid

## Abstract

Melioidosis is a severe disease that can affect humans and animals. It is caused by *Burkholderia pseudomallei,* which is an environmental aerobic gram negative bacteria. Despite being endemic in Southeast Asia and Northern Australia, many cases have been reported from different regions, including the Middle East. This is the third imported case of *B. pseudomallei* from Oman in a 46-year-old Indian male with left knee septic arthritis in less than three weeks after arrival in Oman. He underwent open arthrotomy, and his synovial fluid culture grew a bacteria with dry, pink colonies that were oxidase positive, susceptible to amoxicillin–clavulanic, identified by API NE as *B. pseudomallei*, and confirmed by molecular tests. This single case report highlights the urgent need to increase molecular diagnostic capacity and improve public health surveillance while maintaining a one health tripartite approach.

## Introduction

*Burkholderia pseudomallei (B. pseudomallei)* is a gram-negative, small aerobic, oxidase positive, bipolar, motile rod-shaped, saprophytic bacterial pathogen found in soil and water. It is the leading cause of melioidosis, which affects both animals and humans. While there is no reliable data on the exact annual incidences of melioidosis due to severe underreporting, a recent spatial modeling study published in early 2016 estimated that 165,000 (95% credible interval 68,000–412,000) of human cases occur per year worldwide, from which approximately 89,000 (36,000–227,000) people die, reflecting a mortality rate of more than 50%.^1^ This potentially fatal disease can present with acute, subacute, or chronic symptoms. Half of the patients present with pneumonia. Other manifestations could be genitourinary infection, skin infections, primary bacteremia, musculoskeletal infections, mainly septic arthritis and osteomyelitis, and central nervous system involvement.^2,3^

Several risk factors have been associated with melioidosis, including diabetes mellitus (60%), chronic lung disease (27%), renal disease (10%), and alcohol consumption (39%). However, no risk factors have been identified in 20%–36% of cases.^2,4^

*B. pseudomallei* is transmitted through inhalation, direct inoculation, and ingestion. The incubation period varies from days to many years. It is endemic in Northern Australia and Southeast Asia, including but not limited to Thailand, Vietnam, Laos, Cambodia, Malaysia, Indonesia, Singapore, Papua New Guinea, and the Indian subcontinent.^5^ Due to many reasons, it has been isolated and reported from different countries outside the endemic zone. It is considered a hazard group 3 gram negative bacilli, requiring a minimum Containment Level 3 (CL-3) to handle the organism in the laboratory.^6,7^

## Case Report

A 46-year-old Indian male resided in Oman for the last 11 years while working in a kitchen manufacturing company in the industrial area in Barka, South Batinah Governorate. He lived on the company campus and shared a room with two other people. He worked from 8 am to 5 pm, and he had no other jobs.

The patient arrived in Oman on November 8, 2020. He had left his job in March 2020 due to the shutdown of businesses and other challenges of the COVID19 pandemic. He is originally from Maramon, Kerala, where he spent the whole period with his family. He used to practice gardening activities on his small farm. Neither he nor anyone of his family members had developed any symptoms. Also, none of them had any accidents, injuries, or falls during his leave.

After arrival in Oman, he completed 14 days of quarantine as per the national preventive measures for COVID19 in the country.

Then, on November 25, he started to feel mild to moderate pain in his left knee. There was no history of fever, respiratory or urinary symptoms, back pain, and trauma. Also, he had no history of pain in his other joints. He has no history of any sexual exposure. His pain was not associated with morning stiffness. He did not have any family history of rheumatological diseases. He sought medical consultation at the first private medical center on November 28. A basic blood workup was performed, including complete blood count (CBC), blood glucose, and uric acid, but did not reveal any abnormality. The patient received injections of ceftriaxone 1 g, diclofenac 75 mg, dexamethasone 4 mg, once daily for two days, and oral cefixime 400 mg for five days. On November 30, he was also given an intraarticular lidocaine injection diluted with dextrose and distilled water (as prolotherapy) considering osteoarthritis since his CBC was normal. Almost a week later, the patient returned, stating that he had complete relief for four days, but he started experiencing severe pain in his left knee. At this visit, the joint was swollen, and the patient had difficulty walking due to pain. Thus, the patient was referred for expert opinion in another private hospital. Blood investigations were performed again with the following results. C-reactive protein 105.9 mg/L, erythrocyte sedimentation rate 49 ml/hr, hemoglobin 16.9 gm/dl, platelets 375 k/μI, and white blood cell count (WBC) 16.5 K/μL.

**Figure 1.**
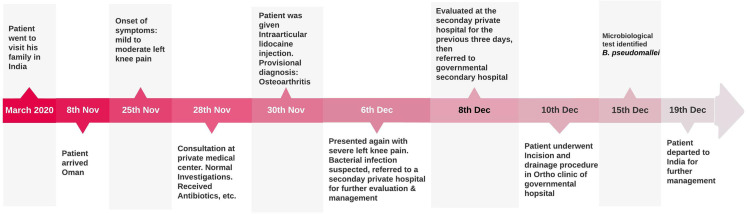
Demonstrates the timeline of the patient's travel history, medical consultations, clinical progress, and tests performed.

**Figure 2.**
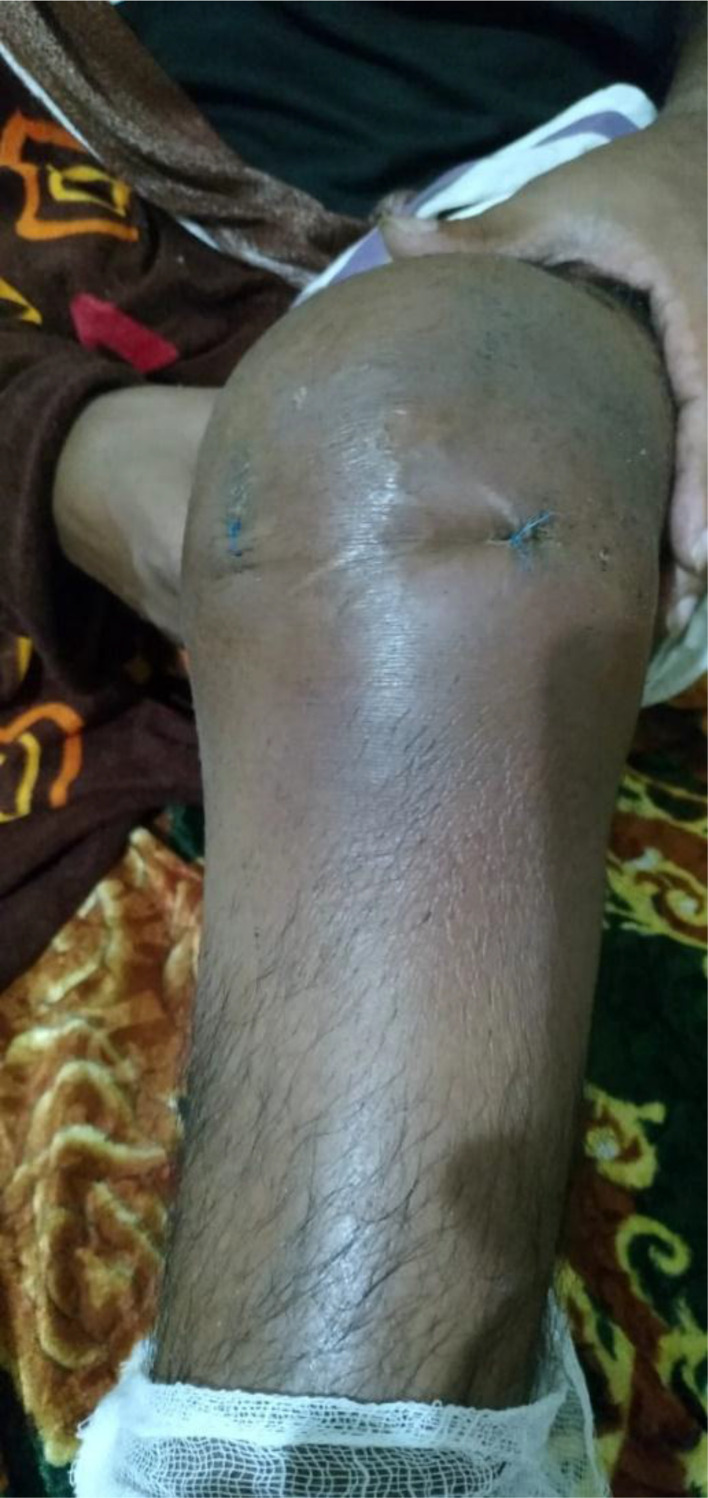
Knee arthrotomy surgery performed in Oman.

Since the results indicated a possible bacterial infection, he received cefditoren pivoxil 200 mg BID for seven days, etoricoxib 90 mg BID for seven days, lansoprazole 30 mg BID for seven days, and injection of diclofenac OD for three days. The patient continued to have severe pain; thus, he was referred to the governmental secondary hospital for further evaluation and management. (Figure 1) He was evaluated in the orthopedic outpatient clinic. His vital signs were normal, and the local examination of his left knee revealed mild joint swelling with erythema, mild tenderness, and increased warmth on palpation. Both active and passive movements elicited his pain. The patient was diagnosed with left knee septic arthritis. Therefore, he underwent urgent open arthrotomy during which synovial fluid mixed with pus and debris was removed. (Figure 2)

**Figure 3.**
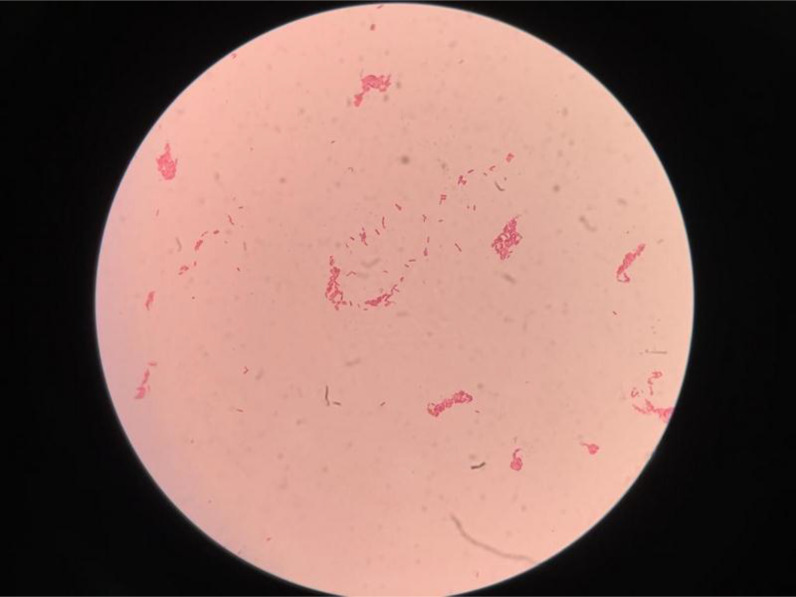
Gram stain: small bipolar Gram-negative bacilli

**Figure 4.**
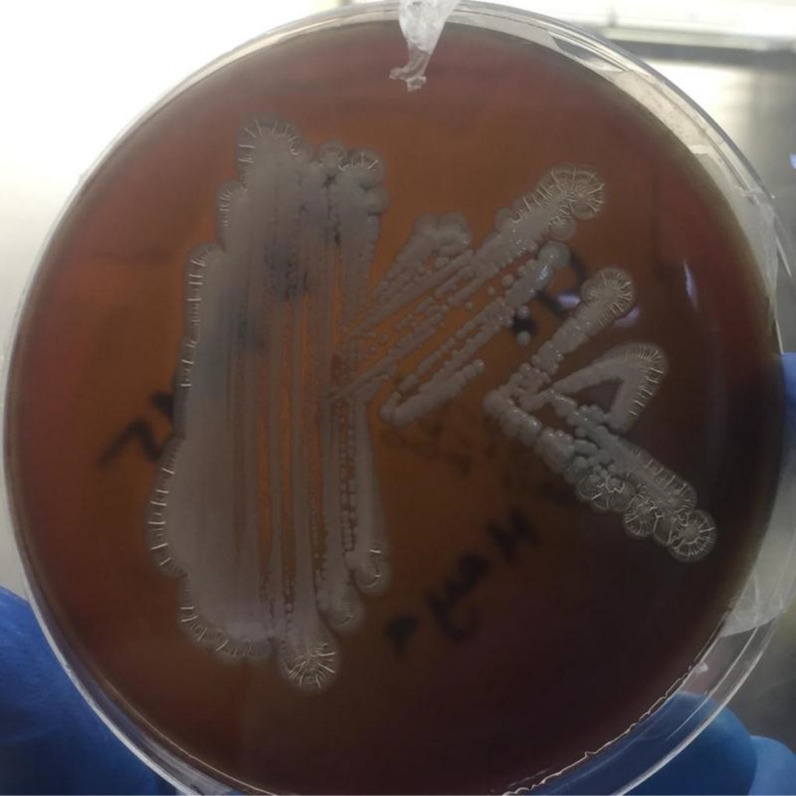
Blood agar: dry white colonies

**Figure 5.**
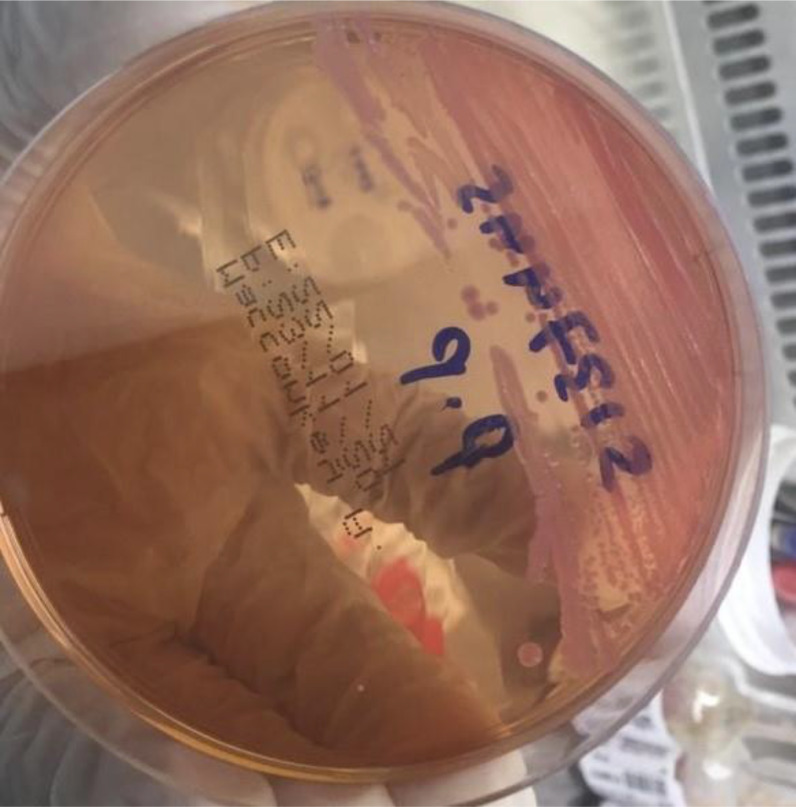
MacConkey agar: round pink colonies

The routine analysis showed a high WBC 70,200  ×  10^6^/L (80% neutrophils/20% lymphocytes). The sample was inoculated on blood agar and MacConkey plates (incubated in ambient air at 37°C). Growth revealed pinkish colonies seen as gram negative bipolar bacilli when Gram-stained and tested positive for oxidase. (Figures 3,4 & 5) API NE (bioMérieux, France) identified *Burkholderia cepaia* with 94% confidence. The antibiotic susceptibility testing by disk diffusion (Oxid, UK) revealed resistance to multiple antibiotics, including aminoglycosides (gentamicin and amikacin), ampicillin, and ciprofloxacin, and susceptibility to amoxicillin–clavulanic acid, ceftazidime, piperacillin–tazobactam, carbapenems, and cotrimoxazole. Due to the discrepancy between colony morphology and susceptibility pattern along with API NE identification and Gram stain appearance, the isolate was sent to Central Public Health Laboratories (CPHL), Bacteriology Section, in Capital City for further identification. The MALDI-TOF (Maldi Biotyper MBT Compass 4.1.100, Bruker Daltoniks GmbH, Germany) identified it as *Burkholderia thailandensis*. Molecular analysis (16 rRNA) identified it as *B. pseudomallei.*

The diagnosis and management were discussed with the patient. However, he preferred to get treated in his country because of financial reasons. The patient was discharged from the hospital on December 14 with ciprofloxacin 500 mg BID and diclofenac sodium 50 mg BID for seven days. On December 19, the patient departed to India for further treatment.

**Figure 6.**
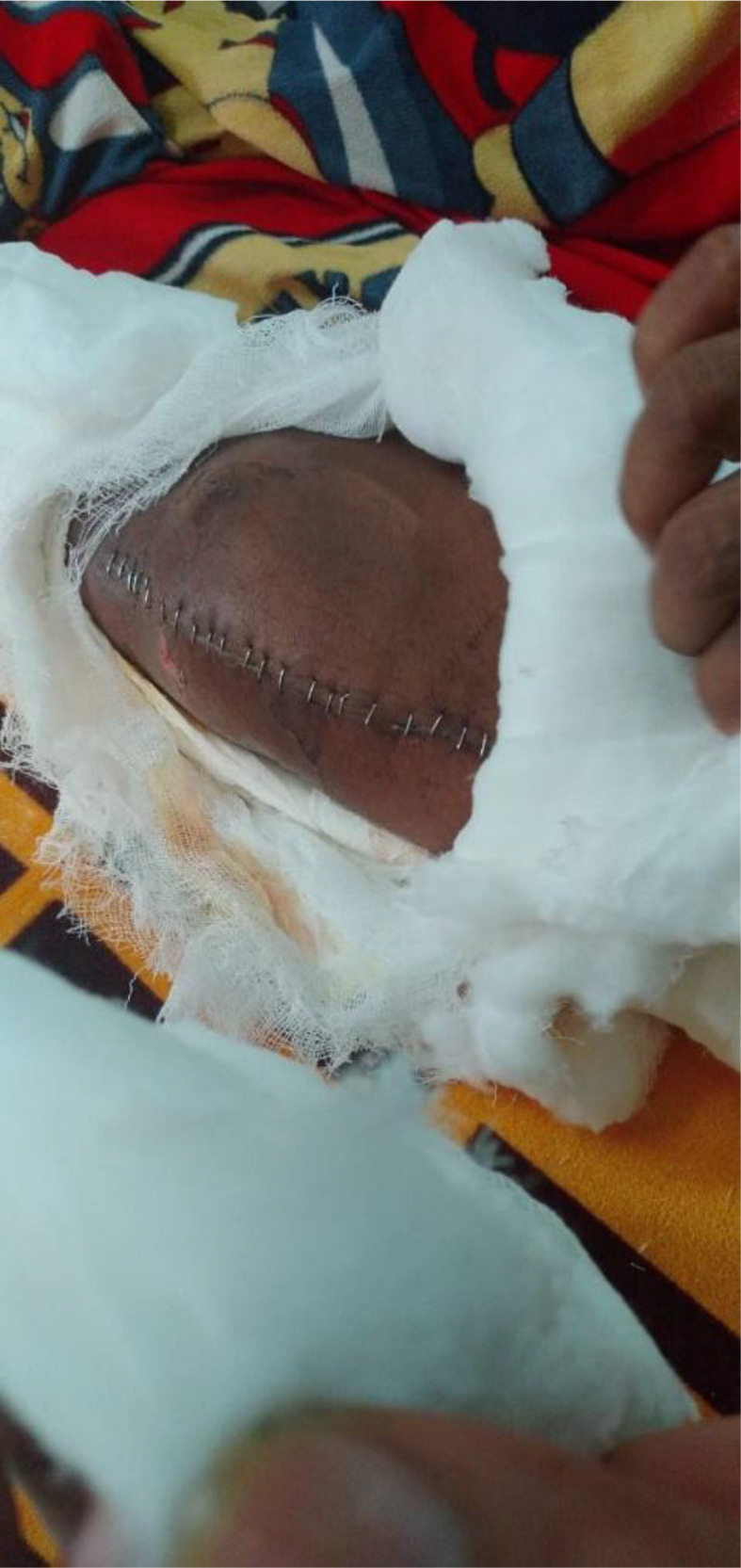
Open knee surgery performed in India

Upon arrival in India, he was readmitted to a hospital where he underwent urgent open knee surgery.(Figure 6) He remained in the hospital for less than three months but had to be discharged home as the hospital was overwhelmed by COVID19 patients. He received meropenem iv and cefuroxime as tablets. His treatment was over in September 2020, and he has finally recovered.

## Discussion

This is the third reported case of melioidosis in Oman as per the Author's knowledge, but the first case with septic arthritis presentation in the country. The first case reported was diagnosed in a previously well, 55-year-old Omani male with a travel history to Laos and Cambodia in 2015. He presented with fever and rigors and was diagnosed with fulminant sepsis. He deteriorated rapidly and died three weeks after ICU admission.^8^ The second case reported was a 47-year-old Sri Lankan patient, who also had diabetes mellitus, and alcohol cirrhotic liver disease. He developed a fever one week before his arrival to Oman in 2019. He was also diagnosed with sepsis and improved after a 14-day course of intravenous meropenem and cotrimoxazole.^9^

Our patient is from India, an endemic area of *B. pseudomallei,* along with other countries in the southeast of Asia.^1,10^ His persistent fever and pain, travel history, and unresponsiveness to the treatment provided by the private medical center alerted the physician of possible infection. Thus he was referred for expert evaluation in a reasonably good time.

Until now, Oman is not considered an endemic area of *B. pseudomallei*. However, a predicting modeling study has speculated that the country might be suitable for growing such bacterial species. There could be unreported cases too similar to neighboring countries, including Saudi Arabia and Yemen.^1^ The study, published in 2016, predicted Oman as probably endemic with melioidosis but never reported it.^1^ Thus, Oman was listed among the prioritizing countries that need to strengthen its microbiological diagnostic facilities and disease reporting systems.

This patient had no known comorbidities; however, melioidosis is common among immunocompromised patients, mainly diabetic patients, especially those with occupational exposure to soil and groundwater in the endemic areas.^11^ Bone and joint involvement are not uncommon, as in our case. Previous studies in northern Australia and northeast Thailand found proportions of rheumatological infections 41/536 (7.6%) and 14% to 27%, respectively.^12–14^

Many diagnostic laboratories outside the endemic countries face difficulty identifying *B. pseudomallei* and have no experience with this species. Due to limited experience, lack of validated diagnostic methods, the presence of closely related species, and atypical colony morphology, identifying *B. pseudomallei* remains challenging in the diagnostic laboratory, even in endemic countries.^15^

In addition, several published papers were written based on the existing epidemiological factors, clinical manifestations, progress, and response to antibiotics. However, none provided a definite microbiological confirmation.^16,17^

Detection of *B. pseudomallei* directly from clinical samples can be easily missed. Although it is a nonfastidious organism and grow easily on most routinely used media in diagnostic labs, it can be misidentified as pseudomonas species, bacillus species, or other closely related Burkholderia species, such as *B. thailandensis* (a phenotypically similar but avirulent species) and members of the *B. cepacia* complex.^18,19^

The isolation of *B. pseudomallei* in culture is the gold standard method. The typical morphology of bipolar staining is seen mainly in a fresh colony and might not be cleared in old growth. The colony appears round, translucent, moist, and slightly raised after 24 hours at 37°C. After 48 hours, they become opaque, dry, and heaped up. MacConkey agar, the colonies are usually pink and dry. Because missing cases or underreporting of *B. pseudomallei* is considered an ongoing challenge in many countries, some studies have focused on the sensitivity of different identification methods rather than specificity. For instance, a study found that the sensitivity of API 20NE, agglutinating antibody, and gas–liquid chromatography analysis of bacterial fatty acid methyl esters (GLC_FAME) methods were 37%, 94%, and 98%, respectively.^20^ However, another study considered API 20NE the most reliable, with an accuracy of 87% and 93% for identifying *B. pseudomallei* and *B. cepacia*, respectively.^21^

The detection of antigens from clinical samples was evaluated using different methods, such as direct Immunofluorescence assays and antibody sandwich ELISAs. Although their sensitivity is significantly lower than culture, their results are helpful diagnostic methods for severely ill patients with high bacterial loads.^19^

Generally, serological tests have low sensitivity and specificity in endemic countries. At the same time, the usage of serological tests outside endemic countries is further complicated due to a lack of evidence and utility. Therefore, they cannot be recommended as routine diagnostic tests.^19^

Using validated PCR tests showed high sensitivity and specificity even from clinical samples. Ultimately, whole-genome sequencing has the highest yield but might not be practical and affordable in all laboratories.^22^

On the other hand, using an automated biochemical system for identifying *B. pseudomallei* poses a particular problem due to low yield and misidentification.^21^

Mass spectrometry identification of organisms has increased over time and is now the standard in many laboratories worldwide. The matrix-assisted laser desorption ionization, time of flight mass spectrometry (MALDI-TOF MS) is used for identifying *B. pseudomallei*. It shows excellent sensitivity and specificity, reduces turn-around time, and enhances antibiotic choice and patient outcome.^23–25^ For instance, a recently published study demonstrated that bioMérieux Vitek MS (bioMérieux, Marcy-l'Etoile, France) could be used for the rapid identification of *B. pseudomallei* with a specificity reaching up to 99.8%.^26^ However, the MALDI-TOF database must be optimized to identify *B. pseudomallei* successfully. In our case, the MALDI-TOF at CPHL did not have *B. pseudomallei* in its database because it was considered a bioterrorism agent and was on a list with restricted access. However, MALDI-TOF commented that it could not differentiate between *B.* thailandensis and *B. pseudomallei*. Such misidentification with B. thailandensis was reported previously using the Bruker Daltonics database.^27^ Therefore, the strain was subjected to 16S rRNA. The limited database of any identification system always needs to be considered.

*B. pseudomallei* has intrinsic resistance to many antibiotics used as first-line treatment of most community-acquired infections, such as penicillin, ampicillin, first- and second-generation cephalosporins, macrolides, rifampicin, and aminoglycosides. The susceptibility profile of the organism enhances the presumptive identification of *B. pseudomallei*, which is sensitive to amoxicillin–clavulanic acid in contrast to *Pseudomonas* species and *B. cepacia* complex.^28^

Despite the abovementioned difficulties, a study from Vietnam demonstrated the possibility of diagnosing many melioidosis cases through a simple and easy-to-perform laboratory algorithm and raising disease awareness at the same time.^29^ The laboratory staff were trained on the typical morphology characteristics of *B. pseudomallei* using different agar media and three antibiotic disc tests (gentamicin, colistin, and amoxicillin/clavulanic acid) as a simple bacterial identification. This easy and inexpensive method helped increase the detection rate of melioidosis cases quickly and at a high specificity level. Although countries still need to develop more sophisticated diagnostic algorithms, this one is more practical in endemic areas and where resources are limited.^29^ The patient presented in this report would have met the suspected case definition in the Vietnamese model. His culture was resistant to gentamicin and sensitive to amoxicillin–clavulanic acid, though colistin was not checked.

The challenges explained in the laboratory setting should also be understood in the real transformation of the global epidemiology of melioidosis. *Burkholderia pseudomallei* is also considered a potential bioterrorism agent by the Centers for Disease Control and Prevention (CDC).^30^ Although the disease existed mainly in the South East of Asia and Australia many decades ago, it has spread to other parts of the globe, including Brazil, China, Malawi, and several developed countries.^1^ The reasons for this distribution could be related to the advancement and availability of the diagnostic capacity in these countries, the importation of infected animals, and the immigration of thousands of immigrants from endemic areas. The risks imposed to Oman seem common among other Gulf countries, which have also reported cases of melioidosis in animals and patients with travel history, mainly to India.^16,17,31,32^

The alert staff in the private and governmental medical sectors played a major role in the successful diagnosis of this case. In addition, the well-established public health laboratory in the country made a considerable effort. This also adds to the importance of maintaining a high level of well-trained and vigilant staff who are always thinking about such uncommon cases, especially among the high-risk groups, while continuously upgrading the diagnostic capacity and implementing the most sophisticated methods. Community engagement is also important by raising the awareness about the disease's symptoms and risk factors for people visiting or coming from endemic countries.

## Conclusion

In conclusion, there is an urgent need to strengthen the surveillance and diagnostic capacities in Oman and the rest of the Gulf countries concerning melioidosis. People from endemic countries require an increased focus to detect and treat cases rapidly. The few reported cases in the Gulf countries may indicate the existence of other potentially undetected melioidosis cases and that the prediction of its transformation into an endemic area will become true.

Declarations:

A. Availability of data and material: Clinical documents of the patient are available on reasonable request.
B. Ethics statement: Written informed consent was obtained from the patient for publication of this case report.
C. Competing interests: The authors declare that they have no competing interests.
D. Funding: No funding was received.
E. Authors’ contributions. Conceptualization: MYS, ZKM, MHS. Data curation: MYS, ZKM, MHS. Funding acquisition: None. Project Supervision: ZKM. Writing - original draft: MYS, ZKM, MHS. Writing - review & editing: MYS, ZKM, MHS, AMJ, AZR.

## Acknowledgment

We wish to thank Dr. Jishan Faisal Chowdhury from Al Burooj Medical Centre and Mr. Noaman Al Hattali form Disease Surveillance and Control Department for their great support, efforts and dedication in the field investigation of this case. In addition, we greatly appreciate the hard work done at Microbiology laboratory in Rustaq Hospital and Central Public Health Laboratory in Muscat.
